# Chemical Constituents from *Mentha haplocalyx* Briq. (*Mentha canadensis* L.) and Their α-Glucosidase Inhibitory Activities

**DOI:** 10.1007/s13659-019-0207-0

**Published:** 2019-04-29

**Authors:** Xiao-Feng He, Chang-An Geng, Xiao-Yan Huang, Yun-Bao Ma, Xue-Mei Zhang, Ji-Jun Chen

**Affiliations:** 1 State Key Laboratory of Phytochemistry and Plant Resources in West China, Kunming Institute of Botany, Chinese Academy of Sciences, Kunming, 650201 People’s Republic of China; 2 Yunnan Key Laboratory of Natural Medicinal Chemistry, Kunming, 650201 China; 3 University of Chinese Academy of Sciences, Beijing, 100049 China

**Keywords:** *Mentha haplocalyx* Briq. (*Mentha canadensis* L.), Lamiaceae, α-Glucosidase inhibitor

## Abstract

**Abstract:**

*Mentha haplocalyx* (*Mentha canadensis*) is widely used as a medicinal plant in traditional Chinese medicine, and the extracts of its aerial parts are found to significantly inhibit the activity of α-glucosidase with an IC_50_ value of 21.0 μg/mL. Bioactivity-guided isolation of the extracts afforded two new compounds (**1** and **2**), together with 23 known ones (**3**–**25**). Their structures were established by extensive spectroscopic analyses (1D and 2D NMR, MS, IR and UV). Compounds **1**–**17** and **21**–**25** were evaluated for their α-glucosidase inhibitory activities. Compound **11** was the most active ones with an IC_50_ values of 83.4 μM. These results verify the α-glucosidase inhibitory activity of *M. haplocalyx* (*M. canadensis*) and specify its active compounds for the first time.

**Graphical Abstract:**

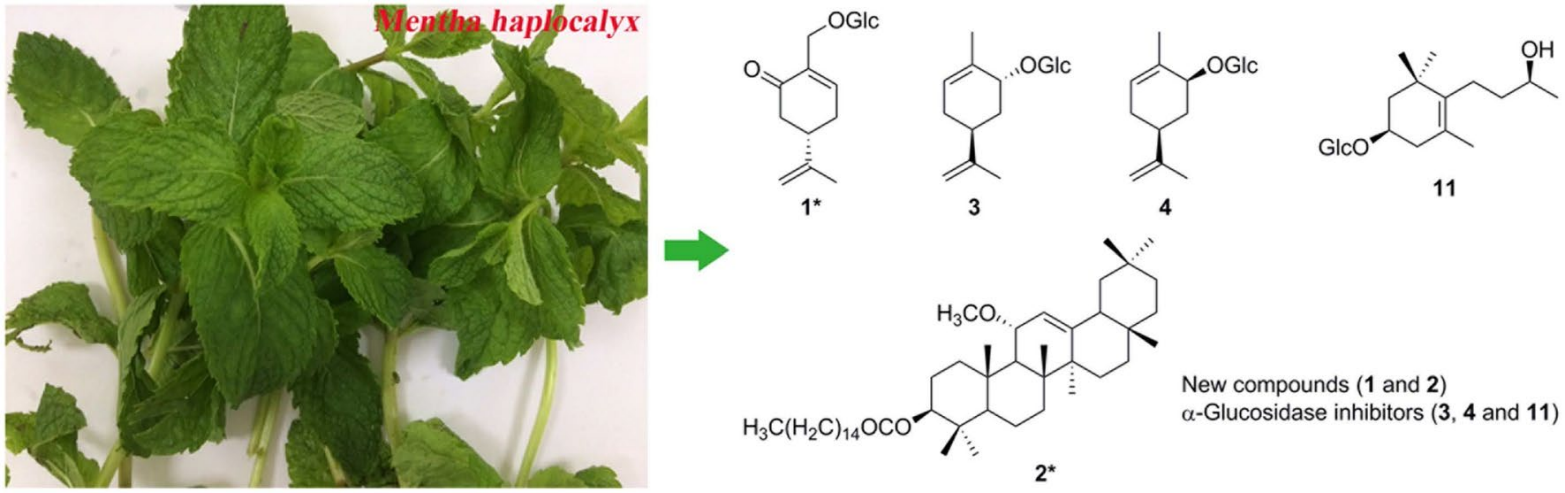

**Electronic supplementary material:**

The online version of this article (10.1007/s13659-019-0207-0) contains supplementary material, which is available to authorized users.

## Introduction

Diabetes mellitus is a chronic disease caused by inherited or acquired deficiency in insulin secretion and by decreased responsiveness of the organs to secreted insulin [1]. Such a deficiency results in an increased blood glucose level and in turn damages body systems including blood vessels and nerves [2]. It is one of the most serious diseases worldwide and developing with an increase in obesity and ageing [3]. An efficiently therapeutic approach is to retard absorption of glucose through the inhibition of carbohydrate-hydrolysing enzymes such as α-amylase and α-glucosidase in the digestive organs [4]. Clinically, acarbose and voglibose have been used as effective α-glucosidase inhibitors to delay glucose absorption [5].

*Mentha haplocalyx* Briq. (*Mentha canadensis* L.), a perennial herbaceous plant of the family Lamiaceae, is widely distributed in southwest of China and popularly used in food, cosmetics and medicines. As a traditional Chinese medicine, it is clinically used to treat diseases in the nerve center, breath, procreation and digestive systems [6]. Pharmacological studies of *M. haplocalyx* (*M. canadensis*) revealed various biological activities, such as antimicrobial, anti-inflammatory, antioxidant, antitumor, gastrointestinal protective, and hepatoprotective activities [7]. A large number of volatile compounds were reported from *M. haplocalyx* (*M. canadensis*), as well as a few polyphenolic acids, flavonoids, monoterpenoids, and glycosides, which might contribute to the medicinal benefits of this plant [8].

**Fig. 1:**
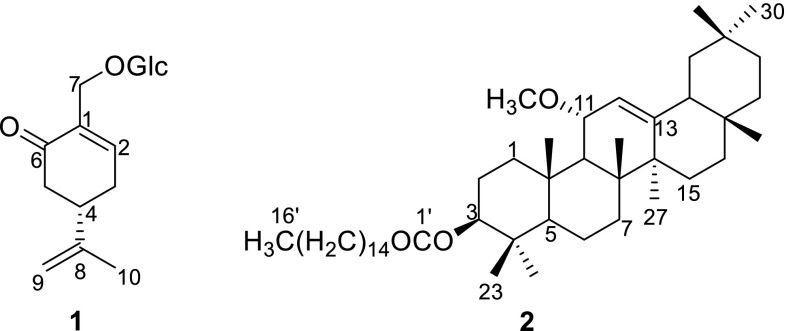
The structures of new compounds **1** and **2** from *M. haplocalyx*

Several findings have depicted the potential antidiabetic capability of genus *Mentha*. *M. piperita* could alleviate hyperglycemia induced by streptozotocin–nicotinamide-induced type 2 diabetes in rats, and cause a reduction of glycemia in human [9, 10]. However, no report has referred to the active compounds of *Mentha* responsible for its antidiabetic capability. Our preliminary bioassay revealed that extracts of the aerial parts of *M. haplocalyx* (*M. canadensis*) exhibited significant α-glucosidase inhibitory activity with an IC_50_ value of 21.0 μg/mL. Subsequently, two new compounds (**1** and **2**) (Fig. 1) and 23 known ones (**3**‒**25**) were isolated and identified through bioactivity-guided fractionation. This paper described the isolation, identification, and α-glucosidase inhibitory activity evaluation of these compounds.

## Results and Discussion

### Structural Identification

**Fig. 2:**
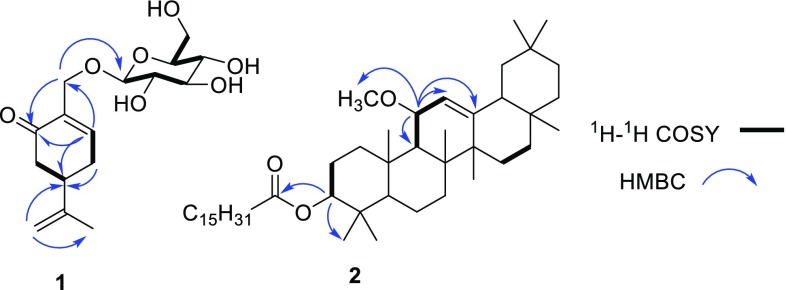
Key ^1^H–^1^H COSY and HMBC correlations of compounds **1** and **2**

Compound **1** was obtained as colorless caramelized solid, with the molecular formula of C_16_H_24_O_7_ from its HRESIMS at *m/z* 351.1404 [M+Na]^+^. The IR absorptions suggested the existence of a hydroxyl group (3427 cm^−1^) and an α,β-unsaturated carbonyl group (1635 cm^−1^). The ^13^C NMR (DEPT) data (Table 1) displayed sixteen carbon signals, including one methyl, five methylenes, seven methines, and three quaternary carbons. Among them, a glucose unit (*δ*_C_ 104.1, 75.2, 78.2, 71.7, 78.2, 62.8) and an α,β-unsaturated carbonyl group (*δ*_C_ 136.6, 149.4, 201.0) were observed. ^1^H NMR data (Table 1) exhibited a singlet methyl proton (*δ*_H_ 1.78, H-10), a trisubstituted olefinic proton (*δ*_H_ 7.23, H-2), and one terminal double bond proton (*δ*_H_ 4.82, H-9a; 4.80, H-9b). The glucose unit protons [*δ*_H_ 4.30 (d, *J* = 7.8 Hz, H-1ʹ); 3.19–3.85 (6H, H-2ʹ‒H-6ʹ)] were also present. All the above information suggested that compound **1** was a menthane-monoterpene glycoside. Apart from the glucose unit, the NMR data of **1** closely resembled those of the reported compound (*R*)-7-hydroxycarvone [11]. Whereas the major differences between their NMR signals at C-7 position [*δ*_H_ 4.49, 4.28 and *δ*_C_ 67.0 for **1**; *δ*_H_ 4.27 and *δ*_C_ 61.8 for (*R*)-7-hydroxycarvone] suggested that 7-OH in **1** might be glycosylated, which was further confirmed by the HMBC correlation of H-7 (*δ*_H_ 4.99 and 4.28) with C-1ʹ (*δ*_C_ 104.1) (Fig. 2). The specific rotation of **1** ($\left[ \alpha \right]_{\text{D}}^{20}$ − 22.0) was opposite to (4*R*)-7-hydroxyisopiperitenone7-*O*-β-d-glucopyranoside ($\left[ \alpha \right]_{\text{D}}^{20}$ + 53.9°), and similar to the (4*S*)-7-hydroxyisopiperitenone 7-*O*-β-d-glucopyranoside ($\left[ \alpha \right]_{\text{D}}^{20}$ − 14.4) [12], tentatively determining its (4*S*)-configuration. Therefore, compound **1** was elucidated as (4*S*)-7-hydroxy-carvone 7-*O*-β-d-glucopyranoside.

**Table 1:** ^1^H NMR (600 MHz, CD_3_OD) and ^13^C NMR (150 MHz, CD_3_OD) data for compound **1**

No	*δ*_H_	*δ*_C_	^1^H–^1^H COSY	HMBC
**1**		136.6 s		
**2**	7.23 m	149.4 d	H-3a, 3b, 7a, 7b	C-1, 3, 4, 6, 7
**3a**	2.67 m	32.2 t	H-2, 3b	C-1, 2, 4, 5
**3b**	2.41 ddq (18.0, 10.2, 1.8)		H-2, 3a	C-1, 2, 4, 5
**4**	2.74 ddd (16.2, 10.2, 4.8)	43.6 d	H-5a, 5b	C-3, 5, 6, 8, 9, 10
**5a**	2.50 ddd (16.2, 5.4, 1.2)	44.2 t	H-4	C-3, 4, 6, 8
**5b**	2.48 m		H-4	C-3, 4, 6, 8
**6**		201.0 s		
**7a**	4.49 dq (13.2, 1.2)	67.0 t	H-7b	C-1, 2, 6, 1ʹ
**7b**	4.28 dd (13.2, 1.2)		H-7a	C-1, 2, 6, 1ʹ
**8**		148.3 s		
**9a**	4.82 m	111.3 s	H-10	C-4, 8, 10
**9b**	4.80 m			C-4, 8, 10
**10**	1.78 s	20.7 q	H-9a	C-4, 8, 9
**1′**	4.30 d (7.8)	104.1 d	H-2ʹ	C-7, 2ʹ, 3ʹ, 5ʹ
**2′**	3.19 dd (9.6, 8.4)	75.2 d	H-1ʹ, 3ʹ	C-3ʹ
**3′**	3.34 t (9.0)	78.2 d	H-2ʹ	C-2ʹ, 4ʹ
**4′**	3.24 m	71.7 d	H-5ʹ	C-5ʹ, 6ʹ
**5′**	3.28 t (9.6)	78.2 d	H-4ʹ, 6ʹa, 6ʹb	C-1ʹ, 4ʹ
**6′a**	3.85 dd (12.0, 1.8)	62.8 t	H-5ʹ, 6ʹb	C-4ʹ, 5ʹ
**6′b**	3.66 dd (12.0, 5.4)		H-5ʹ, 6ʹa	C-4ʹ, 5ʹ

Compound **2**, white powder, had a molecular formula of C_47_H_82_O_3_ from its HREIMS (*m/z* 694.6258 [M]^+^). The ^13^C NMR (DEPT) data (Table 2) displayed 47 carbon signals, including ten methyls, 23 methylenes, six methines and eight quaternary carbons. Among them, a methoxyl (*δ*_C_ 53.4), two oxy-methines (*δ*_C_ 80.4, C-3; 75.8, C-11), a trisubstituted double bond (*δ*_C_ 121.8, 149.7) were showed. A palmitoyl group (*δ*_C_ 173.7, C-1′; 34.7, C-2′; 25.2, C-3′; 29.2–29.7, C-4′–C-13′; 31.9, C-14′; 22.7, C-15′; 14.1, C-16′) was also present. The ^1^H NMR data (Table 2) exhibited eight singlet methyls (*δ*_H_ 0.85–1.21), a triplet methyl (*δ*_H_ 0.88, t, *J *= 7.8 Hz), a methoxyl (*δ*_H_ 3.25), and a trisubstituted olefinic proton (*δ*_H_ 5.32, d, *J *= 3.0 Hz, H-12). These NMR data suggested that **2** was an oleanane-type triterpene fused with a palmitoyl group, and closely resembled those of the reported compound (3β,11α)-11-hydroxy-olean-12-en-3-yl palmitate [13]. The major difference was that the NMR signals at C-11 of the known compound (*δ*_H_ 4.50; *δ*_C_ 81.6) were upfield shifted in **2** (*δ*_H_ 3.89; *δ*_C_ 75.8) and a methoxyl group (*δ*_H_ 3.25; *δ*_C_ 53.4) further appeared, indicating the hydroxyl group at C-11 was methylated in **2**, which was further confirmed by the HMBC (Fig. 2) correlation of OMe (*δ*_H_ 3.25) with C-11 (*δ*_C_ 75.8). Finally, compound **2** was established as (3β,11α)-3-hydroxy-11α-methoxy-olean-12-en-3-yl palmitate.

**Table 2:** ^1^H NMR (600 MHz, CDCl_3_) and ^13^C NMR (150 MHz, CDCl_3_) data for compound **2**

No.	*δ*_H_	*δ*_C_	No.	*δ*_H_	*δ*_C_
**1a**	1.96 m	39.1 t	20		31.1 s
**1b**	1.32 m		21a	1.32 m	34.9 t
**2a**	1.62 m	23.8 t	21b	1.10 m	
**2b**	1.23 m		22a	1.44 m	37.0 t
**3**	4.51 dd (7.8, 9.0)	80.4 d	22b	1.23 m	
**4**		38.0 s	23	0.85 s	28.2 q
**5**	0.88 m	55.3 d	24	0.90 s	16. 8 q
**6a**	1.52 m	18.3 t	25	1.07 s	16.8 q
**6b**	1.26 m		26	1.00 s	18.2 q
**7a**	1.50 m	33.2 t	27	1.21 s	25.1 q
**7b**	1.30 m		28	0.83 s	28.5 q
**8**		43.1 s	29	0.89 s	33.2 q
**9**	1.73 m	51.1 d	30	0.88 s	23.6 q
**10**		38.1 s	OCH_3_	3.25 s	53.4 q
**11**	3.89 dd (3.0, 9.0)	75.8 d	1′		173.7 s
**12**	5.32 d (3.0)	121.8 d	2′	2.29 t (7.8)	34.7 t
**13**		149.7 s	3′	1.64 m	25.2 t
**14**		41.7 s	4′		29.2 t
**15a**	2.04 m	26.3 t	5′		29.3 t
**15b**	0.84 m		6′		29.4 t
**16a**	1.65 m	26.8 t	7′ ~ 13′		29.6 ~ 29.7 t
**16b**	1.00 m		14′	1.26 m	31.9 t
**17**		32.3 s	15′	1.27 m	22.7 t
**18**	2.01 dd (13.8, 4.2)	47.0 d	16′	0.88 t (7.8)	14.1 q
**19a**	1.66 dd (13.8, 4.2)	46.5 t			
**19b**	1.08 m				

By comparing their physical and spectroscopic data with those reported in the literatures, the known compounds (Figure S1) were elucidated as (4*R*,6*R*)-carveol β-d-glucoside (**3**) [14], (4*R*,6*S*)-carveol β-d-glucoside (**4**) [14], (+)-neodihydrocarvy β-d-glucoside (**5**) [15], (−)-dihydrocarvy β-d-glucoside (**6**) [15], uroterpenol β-d-glucoside (**7**) [15], spicatoside A (**8**) [16], spicatoside B (**9**) [16], (3*S*,6*S*)-*cis*-linalool-3,7-oxide (**10**) [17], (3*R*,9*S*)-megastigman-5-en-3,9-diol 3-O-β-d-glucopyranoside (**11**) [18], linarionoside A (**12**) [19], 1,1,5-trimethyl- 6-(3-hydroxyl) cyclohexene-5-yl-1-β-d-pyranoglucoside (**13**) [20], linarionoside B (**14**) [21], (9*S*)-linarionoside B (**15**) [21], (+)-jasmololone glycoside (**16**) [14], (‒)-5′-(β-d-glucopyranosyloxy) jasmonic acid (**17**) [22], maniladiol (**18**) [23], 3β,28-dihydroxy-olean-12-enyl palmitate (**19**) [24], olean-12-ene-28-arboxy-3-palmitate (**20**) [25], ursolic acid (**21**) [26], 1-(β-d-ribofuranosyl)-1H-1,2,4-triazone (**22**) [27], naphthisoxazol A (**23**) [28], menthalactone (**24**) [29], 6-amino-9-[1-(3,4-dihydroxy phenyl)ethyl]-9*H*-purine (**25**) [30], respectively.

### α-Glucosidase Inhibitory Activity

**Fig. 3:**
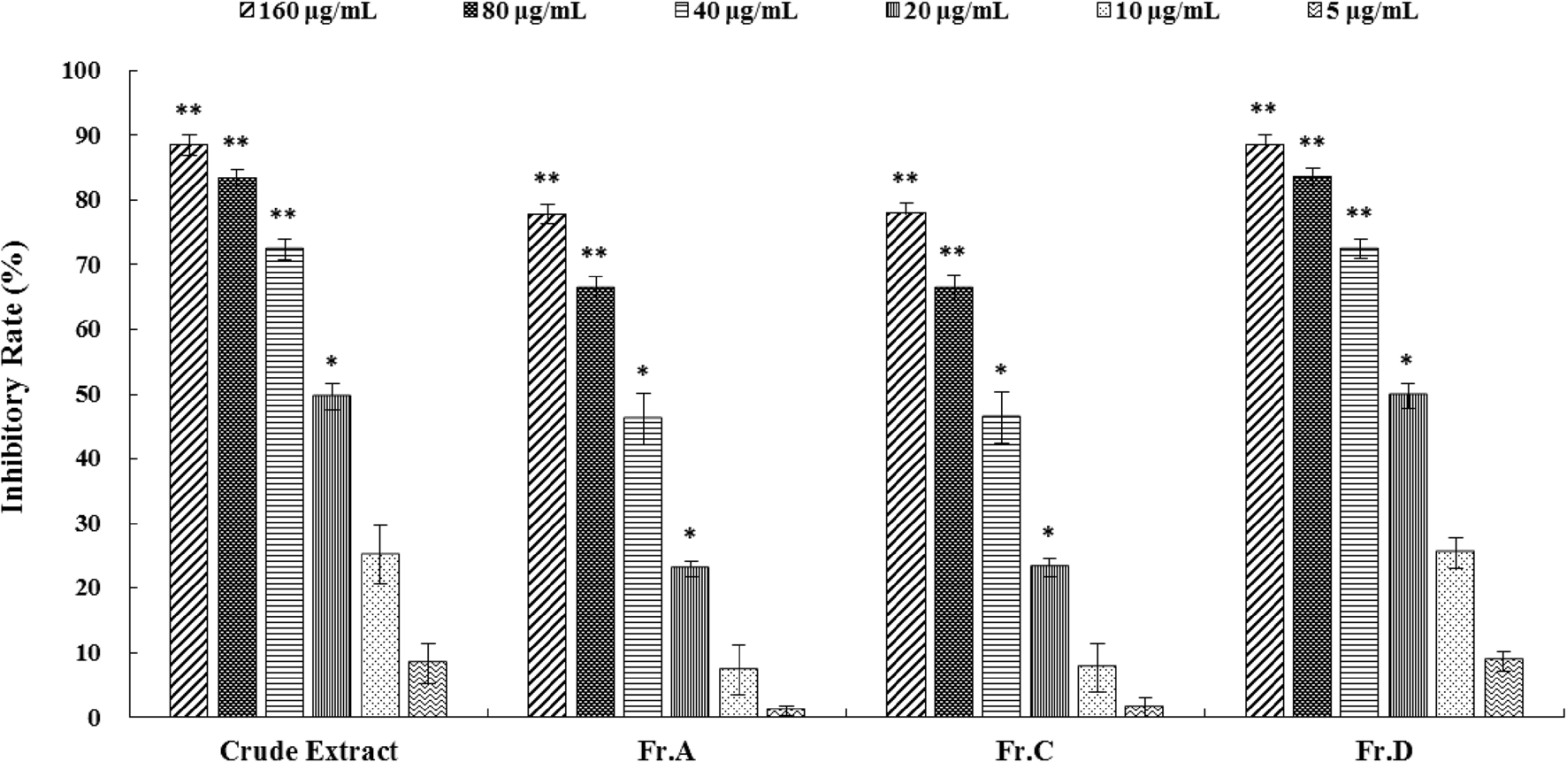
α-Glucosidase inhibitory activities of crude extract, Fr.A, Fr.C and Fr.D at concentrations of 160, 80, 40, 20, 10 and 5 μg/mL, respectively. Values are presented as inhibitory rate compared to the blank control. Means significantly lower than the controls are indicated with one asterisk (*) (Dunnett’s one-sided t test; p < 0.05) or two asterisks (**) (p < 0.01). Error bars are one standard error of the mean. N = 5

In the preliminary bioassay, the crude extracts, Fr.A, Fr.C and Fr.D all exhibited significantly inhibitory activity against α**-**glucosidase at concentrations (> 20 μg/mL). These inhibitory effects were dose-dependent (Fig. 3). Their IC_50_ values were measured as 21.0, 36.7, 37.2, and 20.3 μg/mL, respectively (Table 3). Bioactivity-guided isolation further afforded 25 compounds, while compounds **1**–**17** and **21**–**25** were measured their α**-**glucosidase inhibitory activity. Compound **11** possessed the most significant activity with an IC_50_ values of 83.4 μM, while compounds **3** and **4** showed moderate inhibitory activity against α**-**glucosidase with IC_50_ values of 516.0 and 919.0 μM, respectively (Table 3). Other compounds had no significant inhibitory activity.

**Table 3:** α-Glucosidase inhibitory activities of extracts and compounds from *M. haplocalyx*

Extracts^a^	IC_50_ ± SD	Compounds^b^	IC_50_ ± SD
Crude extract	21.0 ± 1.9	**3**	516.0 ± 2.1
Fr.A	36.7 ± 2.6	**4**	919.0 ± 37.3
Fr.C	37.2 ± 5.5	**11**	83.4 ± 1.3
Fr.D	20.3 ± 2.8		
Acarbose^c^	25.8 ± 3.3		

^a^IC_50_ values in μg/mL are mean ± SD from three independent experiments

^b^IC_50_ values in μM are mean ± SD from three independent experiments

^c^IC_50_ values in nM are mean ± SD from three independent experiments

Comparing the chemical structures and activity of these compounds, it can be found that triterpenoids (**2** and **21**), jasmonoid glucosides (**16** and **17**) and N-containing compounds (**22**–**25**) were not responsible for the α**-**glucosidase inhibitory activity of the extracts from *M. haplocalyx* (*M. canadensis*). Among the monoterpene glucosides (**1** and **3**–**10**), only compounds **3** and **4** manifested moderate inhibitory activity against α**-**glucosidase, while **5** and **6** did not. Thus, it might be assumed that the absence of cyclic double bond in **3** and **4** markedly decreased their inhibitory activity.

## Experimental

### General Experimental Instruments and Procedures

LC–MS analyses were performed on a UFLC/MS-IT-TOF apparatus and the analytical conditions were set as previously reported [31]. Mass spectra were measured through a Waters AutoSpec Premier P776 (Waters, USA) mass spectrometer. Optical rotations were measured through a Jasco model 1020 digital polarimeter (Horiba, Tokyo, Japan). UV and IR (KBr) spectra were recorded on a Shimadzu UV2401PC spectrophotometer (Shimadzu, Kyoto, Japan) and a Bio-Rad FTS-135 spectrometer (Hercules, California, USA), respectively. NMR spectra were recorded on the DRX-500 or AdvanceIII-600 NMR (Bruker, Bremerhaven, Germany) spectrometers with TMS as an internal standard. Thin-layer chromatography (TLC) analyses were carried out with silica gel GF254 (Merck, Chemical Co. Ltd., Shanghai, China), and spots were detected under UV light or by heating after spraying with 10% H_2_SO_4_ in C_2_H_5_OH (v/v). Preparative TLC (PTLC) was purchased from Yantai Jiangyou silicon development Company (Yantai, China). Silica gel (200–300 mesh, Linyi Haixiang Co., Ltd; Linyi, China), Sephadex LH-20 (Amersham Bioscience, Sweden), and D101 macroporous adsorption resin (Tianjin guangfu fine chemical co. LTD, Tianjin, China) were used for column chromatography. MPLC separations were conducted on a Dr-Flash II apparatus using a MCI gel CPH 20P (75-150 u, Mitsubishi Chemical Corporation, Tokyo, Japan) column. Semipreparative HPLC purifications were performed on a Shimadzu LC-CBM-20 system (Shimadzu, Kyoto, Japan) with the XDB-C18 column.

### Plant Material

The aerial parts of fresh *M. haplocalyx* Briq. (*M. canadensis* L.) were bought from market, Kunming, Yunnan Province, China, in March 2013, and were identified by Prof. Li-gong Lei, Kunming Institute of Botany. A voucher specimen (No. 2013032401) was deposited at the Laboratory of Anti-virus and Natural Medicinal Chemistry, Kunming Institute of Botany, CAS.

### Extraction and Isolation

The aerial parts of fresh *M. haplocalyx* (*M. canadensis*) (20 kg) were powdered and extracted with 50% ethanol for three times at room temperature (each 200 L). The combined extraction was concentrated in *vacuo* to yield a residue. The residue was then suspended in water and extracted with ethyl acetate (Fr.A, 130 g). The aqueous phase was subjected to D101 macroporous adsorption resin using a step gradient elution with C_2_H_5_OH-H_2_O (0:100, 50:50 and 90:10, v/v) as the mobile phase to give three fractions (Fr.B–Fr.D).

The Fr.A (130 g) and Fr.D (10 g) were combined (Fr.E, 140 g). Fr.E (140 g) was fractionated on silica gel CC using a step gradient elution with MeOH–CHCl_3_ (2:98, 5:95, 10:90, v/v) and H_2_O–MeOH–CHCl_3_ (2:20:80, 3:30:70. 4:40:60, v/v) as the mobile phase, and following washed with MeOH to give seven fractions (Fr.E-1–Fr.E-7). Fr.E-3 (34 g) was subjected to silica gel CC eluted with EtOAc-petroleum ether (from 6:94 to 50:50) to give five fractions (Fr.E-3-1–Fr.E-3-5). Fr.E-3-1 (8.8 g) was submitted to silica gel CC eluted with Me_2_CO-petroleum ether (5:95), then applied to Sephadex LH-20 CC eluted with CHCl_3_–MeOH (50:50), and finally purified by silica gel CC with EtOAc-petroleum ether (40:60) to yield **2** (10 mg), **19** (13 mg) and **20** (12 mg). Compound **21** (2 g) was got from Fr.E-3-2 (9.1 g) that was separately separated on MCI gel CHP 20P CC (MeOH–H_2_O, 30:70, 70:30, 90:10) and Sephadex LH-20 CC (MeOH–CHCl_3_, 50:50). Fr.E-3-5 (5.6 g) was subjected on MCI gel CHP 20P CC using MeOH–H_2_O (60:40, 90:10), subsequently compound **24** (10 mg) was yielded from Sephadex LH-20 CC (MeOH–CHCl_3_, 50:50). Fr.E-5 (5.8 g) was subjected to MCI gel CHP 20P CC using a step gradient elution with MeOH–H_2_O (40:60, 80:20) to give two fractions (Fr.A-5-1 and Fr.A-5-2). Fr.A-5-1 (2.7 g) was fractionated on silica gel CC (MeOH–CHCl_3_, 10:90) to give three fractions (from Fr.E-5-1-1 to Fr.E-5-1-3). Fr.E-5-1-1 (327 mg) was preferred on Sephadex LH-20 eluted with MeOH–CHCl_3_ (50:50), and following purified by semipreparative HPLC (MeCN–H_2_O, 25:75, v/v, 3.0 mL/min) over an XDB-C_18_ column (9.4 × 250 mm, 5 μm) to yield compounds **3** (5 mg), **5** (4 mg), **6** (5 mg) and **25** (8 mg). Fr.E-5-1-2 (1.3 g) was separated on Sephadex LH-20 CC (MeOH–CHCl_3_, 50:50), and later fractionated on silica gel CC (MeOH–CHCl_3_, 90:10), therefore, compounds **1** (4 mg), **7** (14 mg), **10** (2 mg), the mixture of **12** and **13** (8 mg), the mixture of **14** and **15** (6 mg) were obtained by semipreparative HPLC (MeCN-H_2_O, 40:60, v/v, 3.0 mL/min) over an XDB-C_18_ column (9.4 × 250 mm, 5 μm).

Fr.C (50 g) was subjected to silica gel CC and then Al_2_O_3_ CC with H_2_O–MeOH–CHCl_3_ (3:30:70) to give four fractions (from Fr.C-1 to Fr.C-4). Fr.C-1 (10.4 g) was fractionated on MCI gel CHP 20P CC with MeOH–H_2_O (40:60, 80:20), then applied to Sephadex LH-20 CC eluted with MeOH–CHCl_3_ (50:50), and later purified by silica gel CC eluted with MeOH–EtOAc (2:98) to yield **4** (120 mg), **8** (800 mg), **9** (311 mg), **11** (200 mg) and **16** (20 mg). Compounds **17** (201 mg), **22** (46 mg) and **23** (17 mg) were afforded from Fr.C-2 (20.0 g) by repeated silica gel CC (MeOH–CHCl_3_, 15:85, 20:80, 25:75) and Sephadex LH-20 (MeOH–CHCl_3_, 50:50).

### Spectroscopy Data of Compounds

#### Compound **1**

Colorless caramelized solid, C_16_H_24_O_7_, $\left[ \alpha \right]_{\text{D}}^{20}$ − 22.0 (*c*, 0.20, MeOH); UV (MeOH) *λ*_max_ (log *ε*): 230 (3.65) nm; IR (KBr) *ν*_max_: 3427, 1635, 1385, 1046, 902 cm^−1^; ^1^H NMR (600 MHz, CD_3_OD) and ^13^C NMR (DEPT, 150 MHz, CD_3_OD) see Table 1; HRESIMS *m*/*z*: 351.1404 [M+Na]^+^ (calc. 351.1414 for C_16_H_24_O_7_Na).

#### Compound **2**

Colorless powder, C_47_H_82_O_3_; ^1^H NMR (600 MHz, CDCl_3_) and ^13^C NMR (DEPT, 150 MHz, CDCl_3_) see Table 2; HREIMS *m*/*z*: 694.6258 [M]^+^ (calc. 694.6264 for C_47_H_82_O_3_).

### Inhibitory Assay of α-Glucosidase

The α-glucosidase inhibitory activity was measured in a 96-well microtiter plate based on p-nitrophenyl-α-d-glucopyranoside (PNPG, Yuanye Biosciences Co. Ltd., Shanghai, China) as a substrate following the reported method with slight modifications [32]. In brief, 5.0 mM PNPG (20 μL) and 20 μL tested compounds of dissolved in 10 μL DMSO and 990 μL phosphate buffer (PB, 0.1 M, pH = 6.8) were sequentially added to a 96-well plate to be mixed. The mixture was incubated at 37 °C for 5 min. Reactions were initiated by addition of 2.0 U/mL α-glucosidase (20 μL, Yuanye Biosciences Co. Ltd., Shanghai, China) in PB. The reaction mixture was incubated at 37 °C for 15 min. Then, the incubation solution was stopped the reaction by adding 0.2 M Na_2_CO_3_ (40 μL). The absorbance was recorded at 405 nm by a Bio-Rad 680 microplate reader (Hercules, CA, USA). The negative control was set by adding PB instead of the sample using the same procedure for the tests. Acarbose (Bayer) dissolved in PB was utilized as the positive control. The blank was set by adding phosphate buffer instead of the α-glucosidase using the same method. Inhibition rate (%) = [(ODnegative control − ODblank) − (ODtest − ODtest blank)]/(ODnegative blank − ODblank) × 100%. All data were subjected to an analysis of variance using SPSS 18.0. The significant differences in inhibition rates between the treatment and blank control were calculated using one-way analysis of variance (ANOVA).

## Conclusion

In this study, the extracts of the aerial parts of *M. haplocalyx* (*M. canadensis*) were firstly found to exhibit significantly inhibitory activity against α-glucosidase. Two new compounds (**1** and **2**) and 23 known ones were isolated and identified through bioactivity-guided fractionation. Among them, compounds **3**–**9** and **24** were reported from *M. haplocalyx* (*M. canadensis*) for the first time, while compounds **10**–**20**, **22**–**23** and **25** were firstly isolated from the genus *Mentha*. Bioactivity assay further traced the active compounds (**3**, **4** and **11**), whose inhibitory activity against α-glucosidase had not been reported before. It was noted that the monoterpene glucosides and the ionone glycosides endowed this plant with the α-glucosidase inhibitory activity.

## Electronic supplementary material

Below is the link to the electronic supplementary material.

Supplementary material 1 (DOC 1878 kb). HRMS, IR, UV, 1D and 2D NMR spectra of compounds **1** and **2**; Structures of the known compounds **3**–**25**.
