# Supplementary material for: Chemical Constituents from Mentha haplocalyx Briq. (Mentha canadensis L.) and Their α-Glucosidase Inhibitory Activities
Source: Nat Prod Bioprospect. 2019 Apr 29;9(3):223–9. doi: 10.1007/s13659-019-0207-0 (PMC6538733; doi:10.1007/s13659-019-0207-0)

**Electronic Supplementary Material**

**Chemical constituents from *Mentha haplocalyx* Briq. (*Mentha* *canadensis* L.) and their** **α-glucosidase inhibitory activities**

Xiao-Feng He^a,b,c^, Chang-An Geng^a,b^, Xiao-Yan Huang^a^,^b^, Yun-Bao Ma^a,b^, Xue-Mei Zhang^a,b^ and Ji-Jun Chen^a,b,c *^

*^a^ State Key Laboratory of Phytochemistry and Plant Resources in West China, Kunming Institute of Botany, Chinese Academy of Sciences, Kunming 650201, China*

*^b^ Yunnan Key Laboratory of Natural Medicinal Chemistry, Kunming 650201, China*

*^c^ University of Chinese Academy of Sciences, Beijing 100049, China*

*Corresponding author. Kunming Institute of Botany, Chinese Academy of Sciences, Kunming 650201, P. R. China. Tel.: +86 871 65223265; Fax: +86-871-65227197. *E-mail address*: [chenjj@mail.kib.ac.cn](mailto:chenjj@mail.kib.ac.cn) (Ji-Jun Chen).

**List of Supplementary material**

**Figure S1**. The structures of known compounds **3**–**25**

**S1**. ^1^H NMR (600 MHz, CD_3_OD) of the new compound **1**

**S2**. ^13^C NMR (DEPT) (150 MHz, CD_3_OD) of the new compound **1**

**S3**. ^1^H-^1^H COSY (600 MHz, CD_3_OD) of the new compound **1**

**S4**. HSQC (600 MHz, CD_3_OD) of the new compound **1**

**S5**. HMBC (600 MHz, CD_3_OD) of the new compound **1**

**S6**. ROESY (600 MHz, CD_3_OD) of the new compound **1**

**S7**. IR of the new compound **1**

**S8**. UV of the new compound **1**

**S9**. HRESIMS of the new compound **1**

**S10**. ^1^H NMR (600 MHz, CDCl_3_) of the new compound **2**

**S11**. ^13^C NMR (DEPT) (150 MHz, CDCl_3_) of the new compound **2**

**S12**. ^1^H-^1^H COSY (600 MHz, CDCl_3_) of the new compound **2**

**S13**. HSQC (600 MHz, CDCl_3_) of the new compound **2**

**S14**. HMBC (600 MHz, CDCl_3_) of the new compound **2**

**S15**. ROESY (600 MHz, CDCl_3_) of the new compound **2**

**S16**. HRESIMS of the new compound **2**

**Figure S1**. The structures of known compounds **3**–**25**

**S1**. ^1^H NMR (600 MHz, CD_3_OD) of the new compound **1**


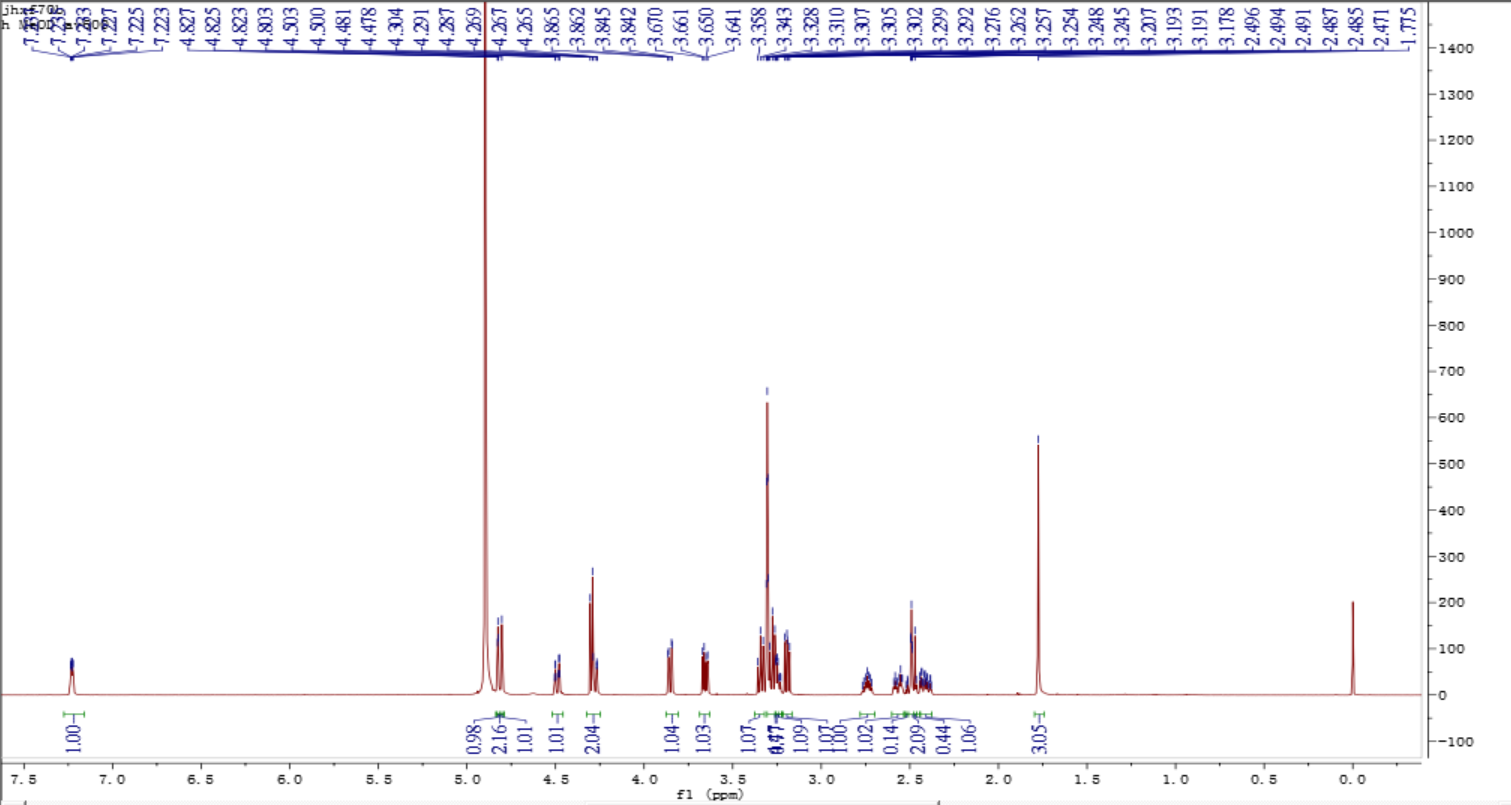


**S2**. ^13^C NMR (DEPT) (150 MHz, CD_3_OD) of the new compound **1**


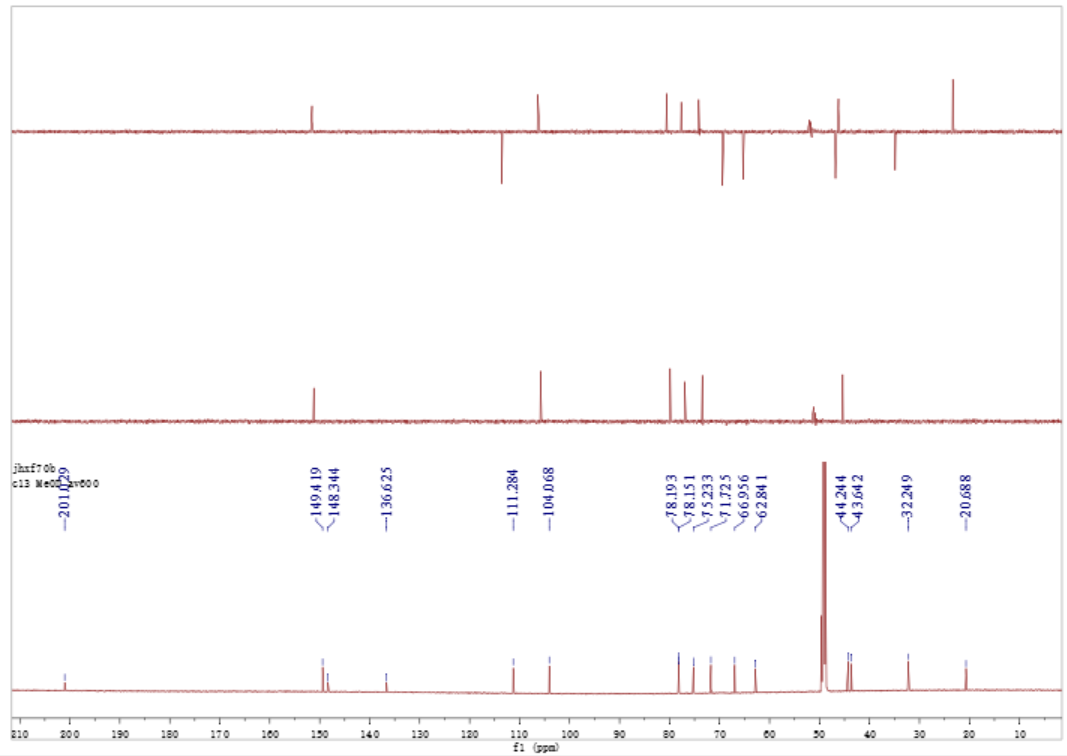


**S3**. ^1^H-^1^H COSY (600 MHz, CD_3_OD) of the new compound **1**


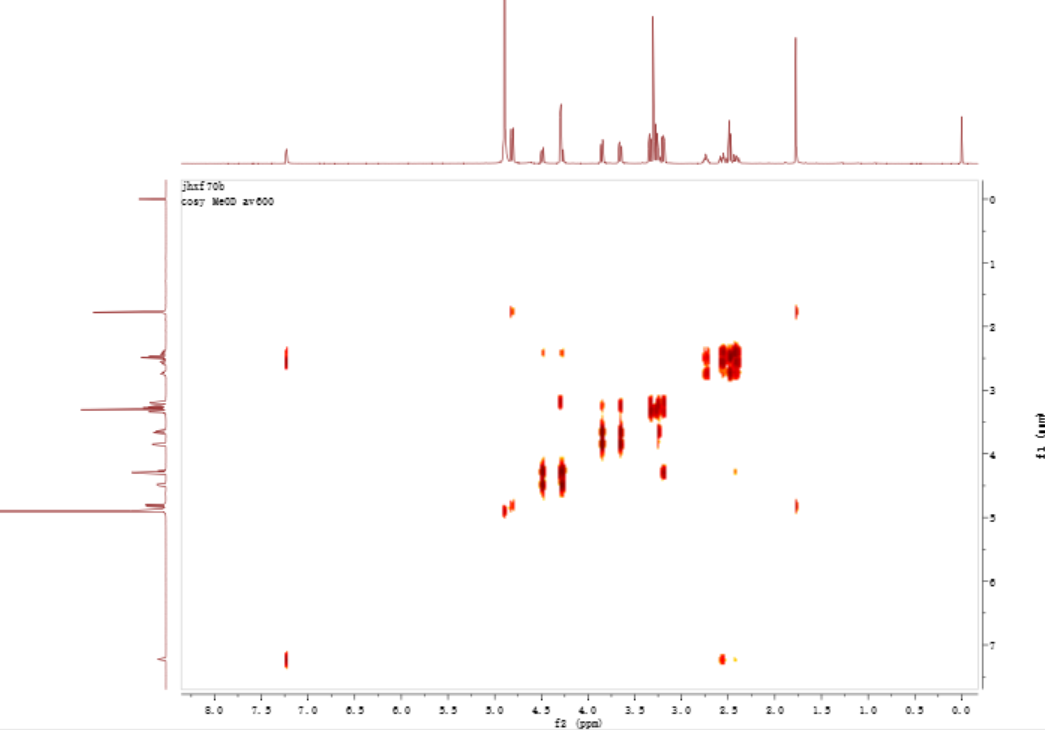


**S4**. HSQC (600 MHz, CD_3_OD) of the new compound **1**


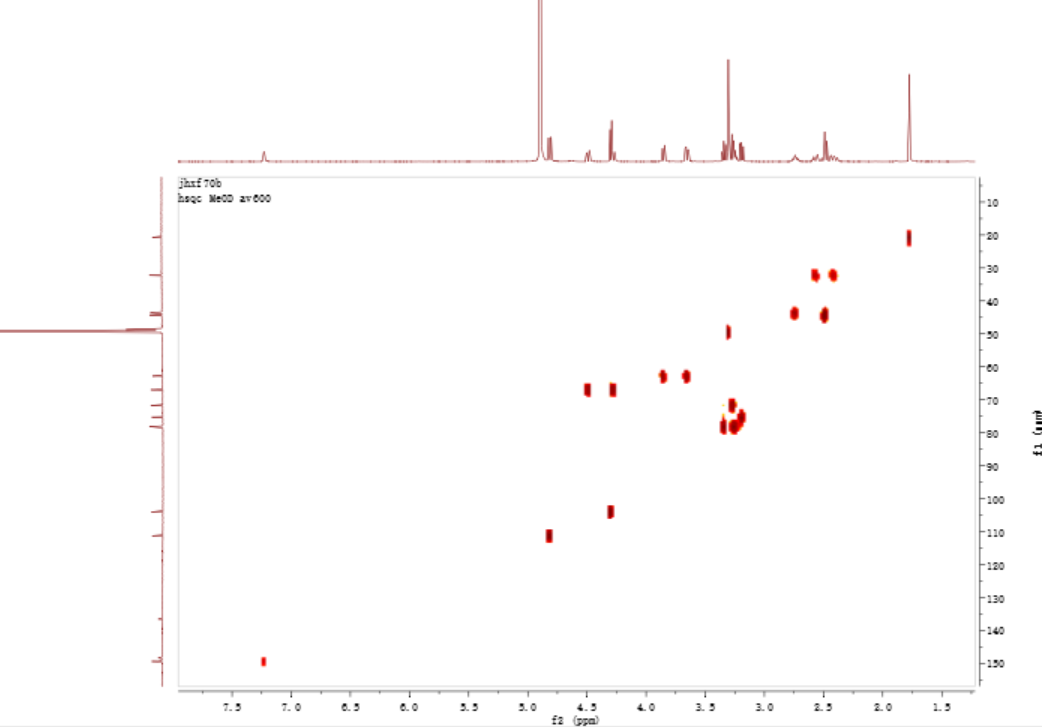


**S5**. HMBC (600 MHz, CD_3_OD) of the new compound **1**


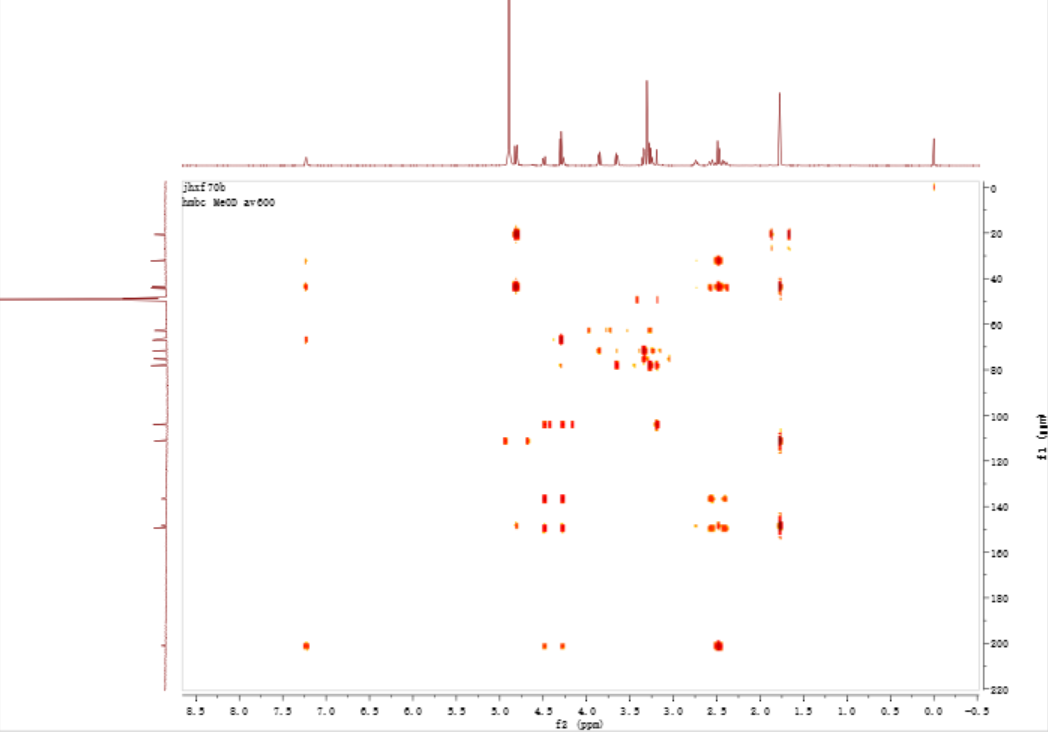


**S6**. ROESY (600 MHz, CD_3_OD) of the new compound **1**


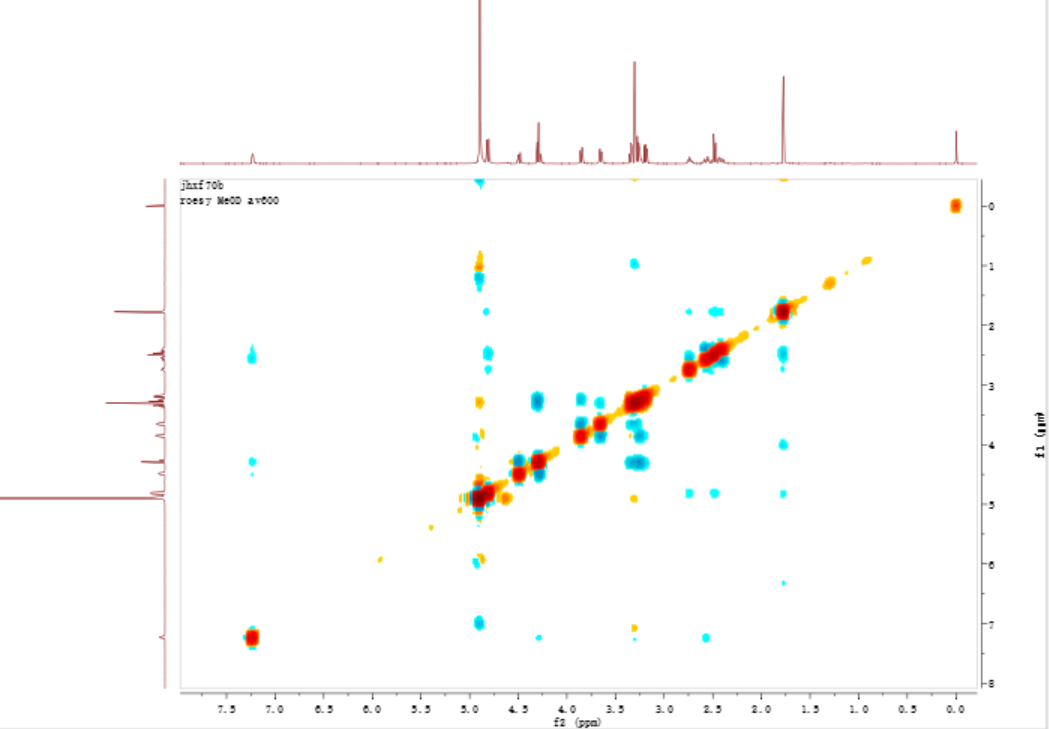


**S7**. IR of the new compound **1**


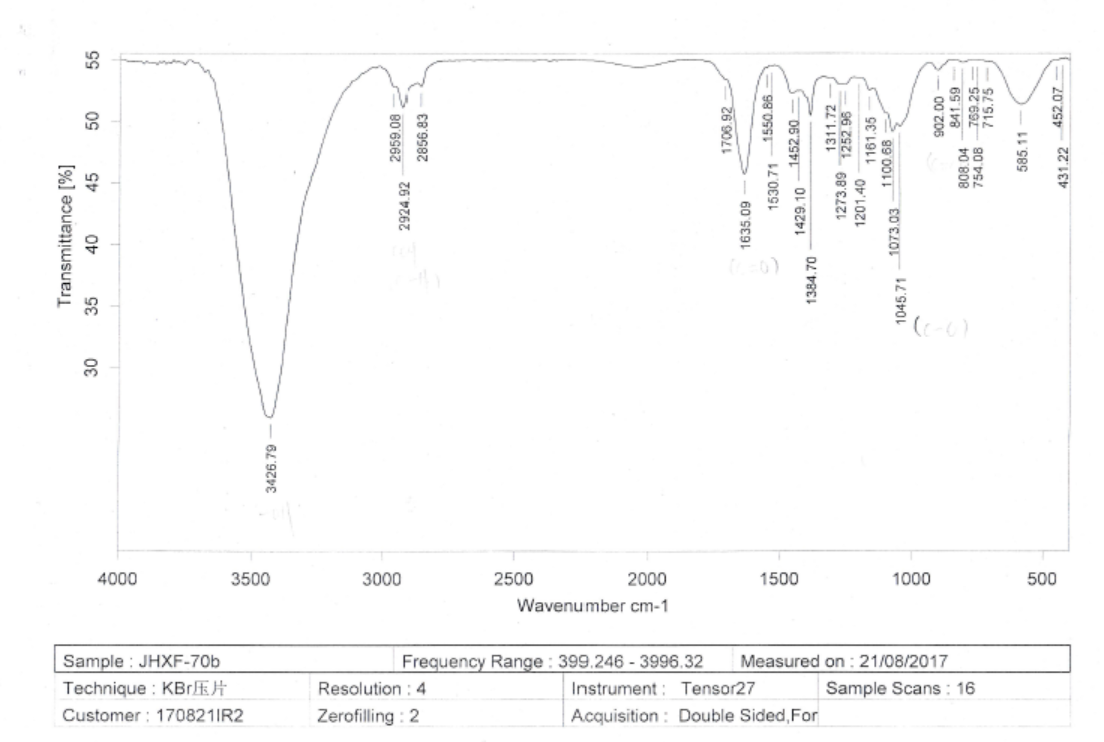


**S8**. UV of the new compound **1**


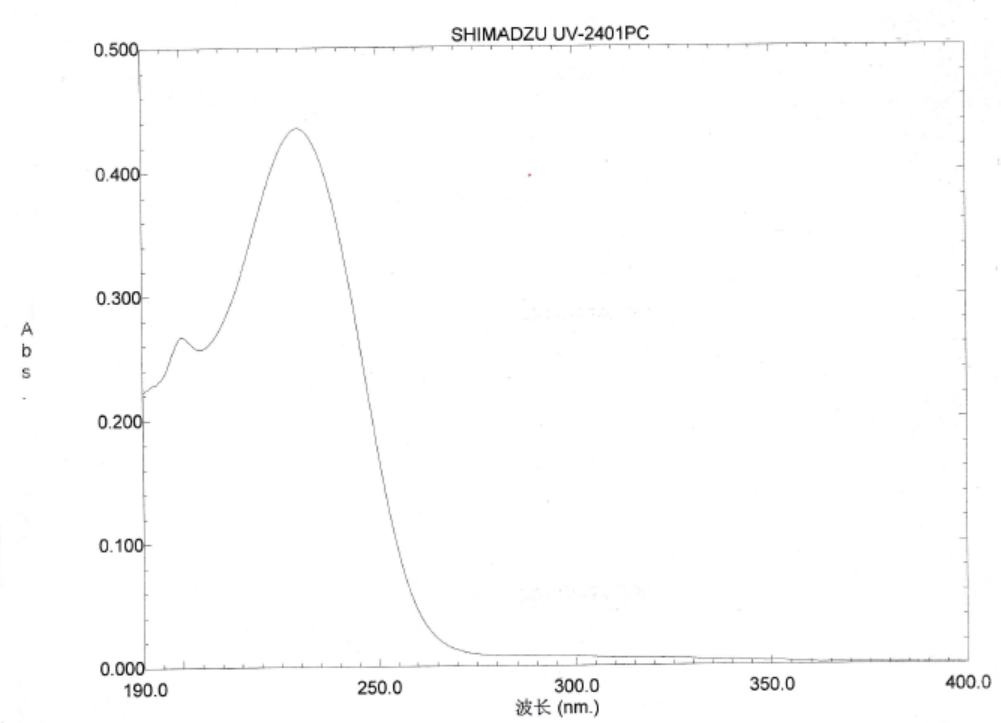


**S9**. HRESIMS of the new compound **1**


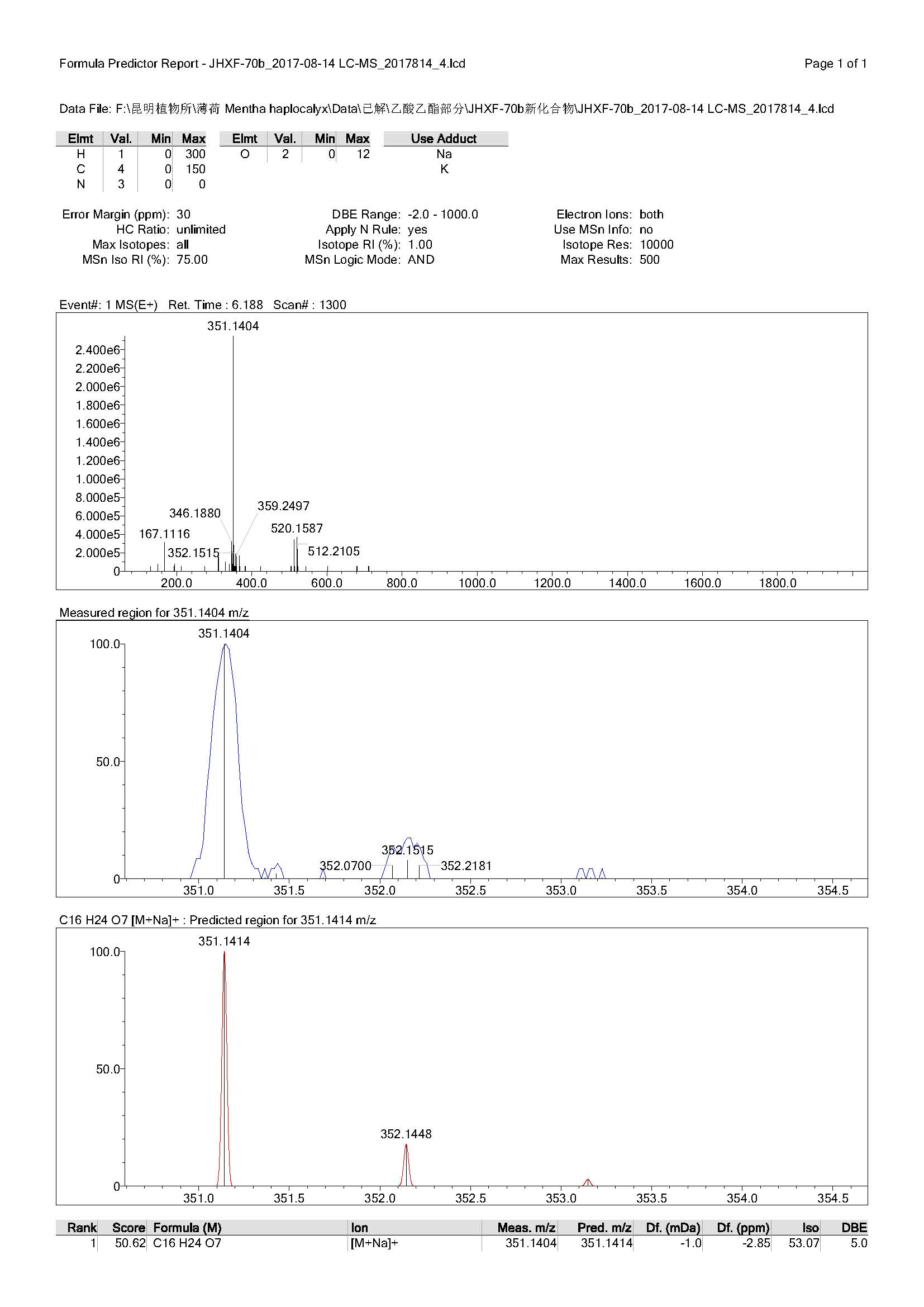


**S10**. ^1^H NMR (600 MHz, CDCl_3_) of the new compound **2**


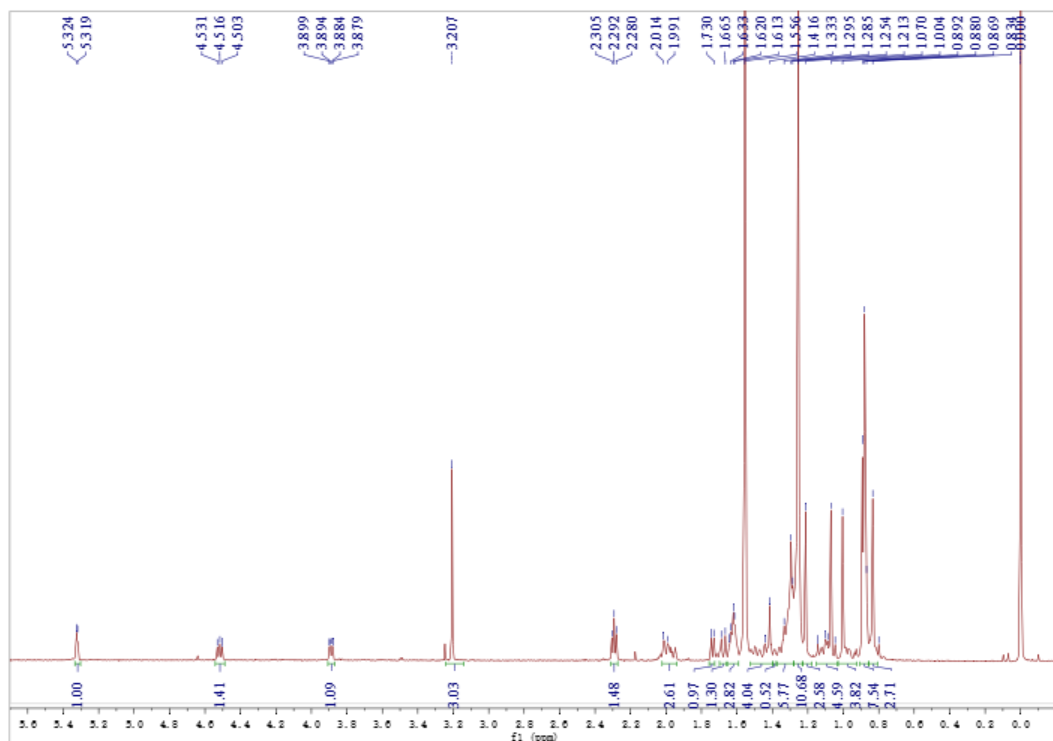


**S11**. ^13^C NMR (DEPT) (150 MHz, CDCl_3_) of the new compound **2**


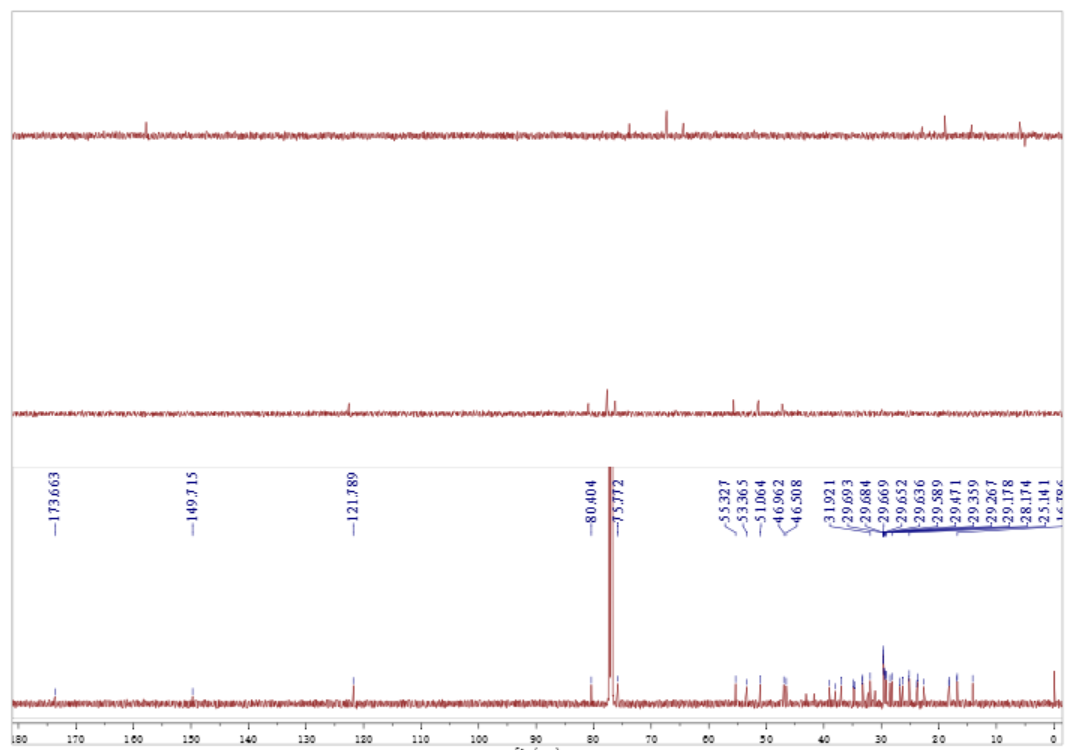


**S12**. ^1^H-^1^H COSY (600 MHz, CDCl_3_) of the new compound **2**


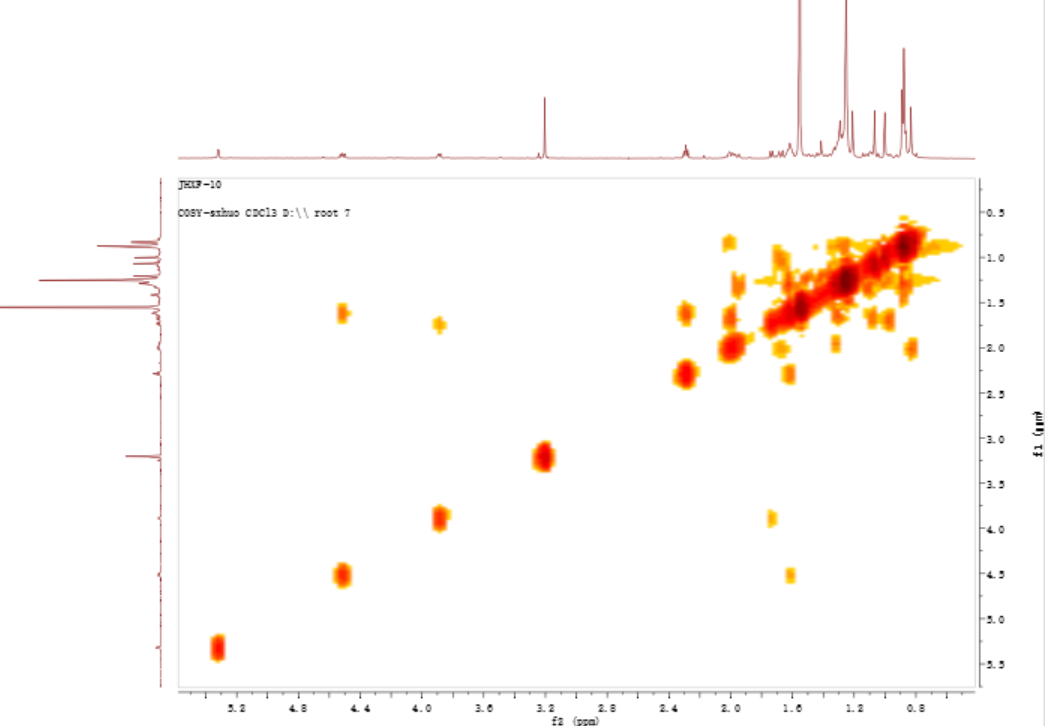


**S13**. HSQC (600 MHz, CDCl_3_) of the new compound **2**


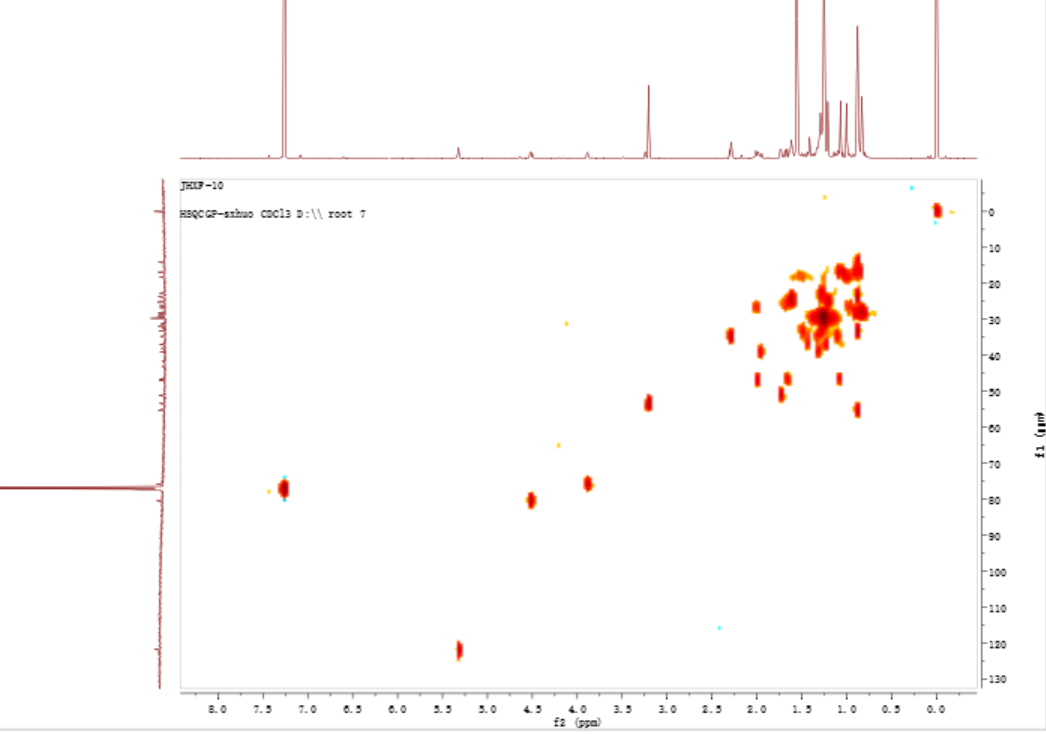


**S14**. HMBC (600 MHz, CDCl_3_) of the new compound **2**


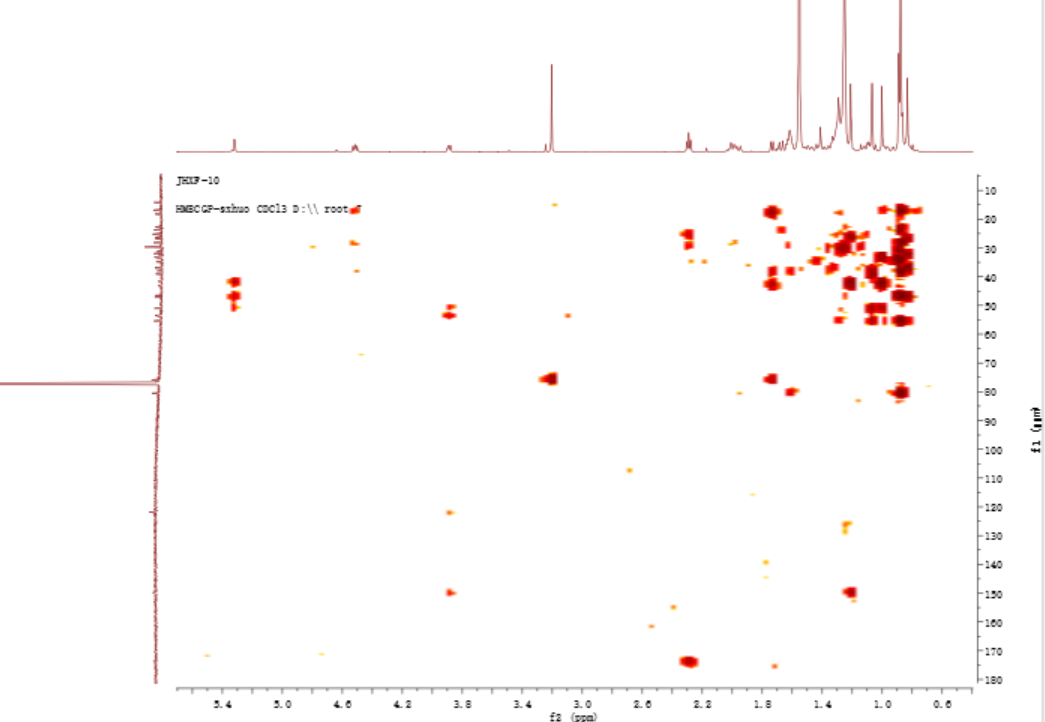


**S15**. ROESY (600 MHz, CDCl_3_) of the new compound **2**


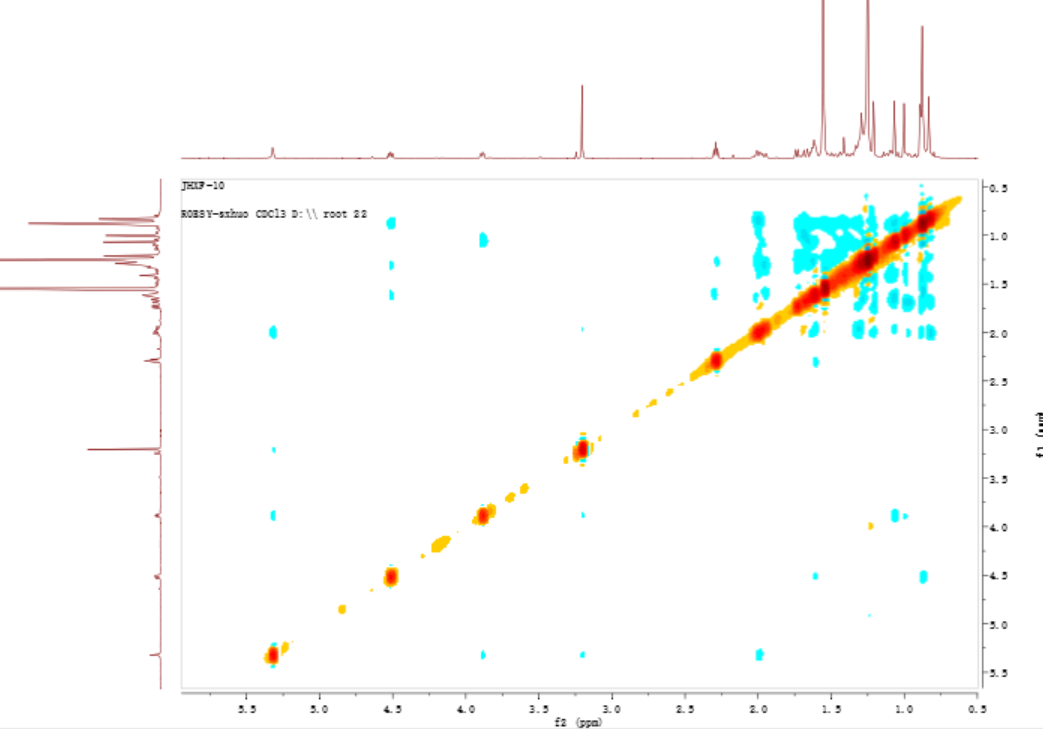


**S16**. HRESIMS of the new compound **2**


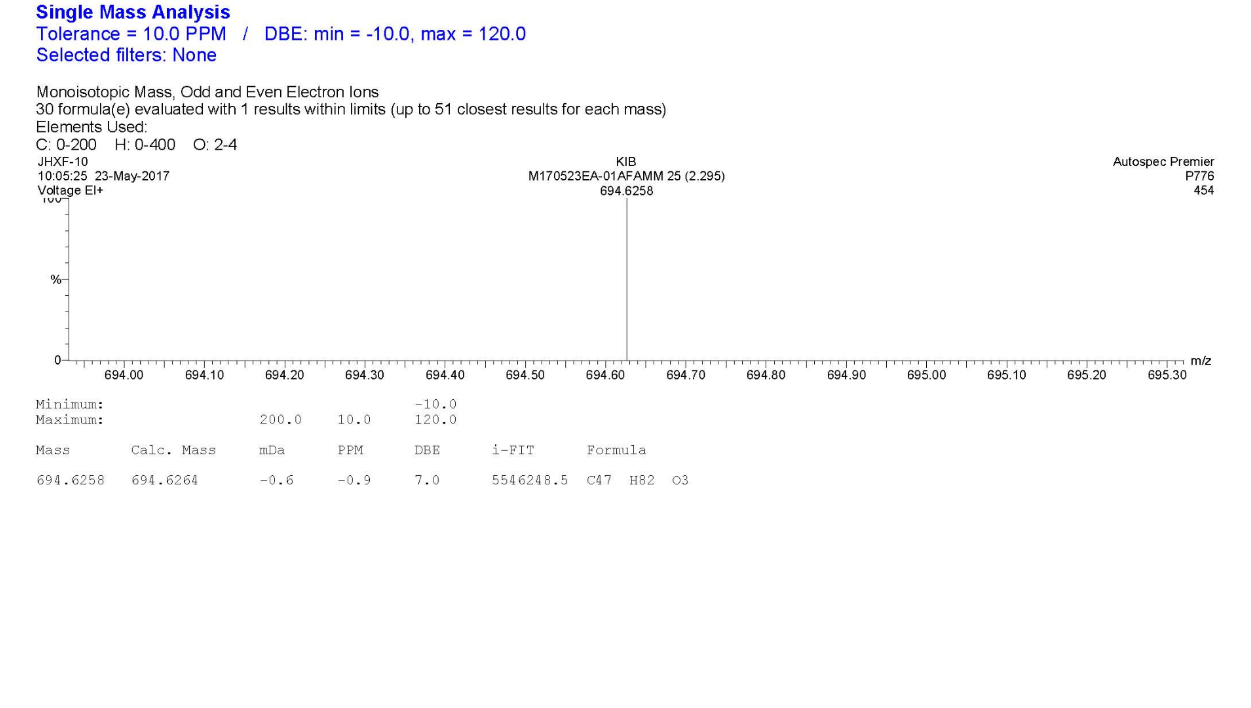

Supplement: Supplementary file 1 — Supplementary material 1 (DOC 1878 kb). HRMS, IR, UV, 1D and 2D NMR spectra of compounds 1 and 2; Structures of the known compounds 3–25. [file 13659_2019_207_MOESM1_ESM.doc]
